# The Fundamentals of Respiratory Physiology to Manage the COVID-19 Pandemic: An Overview

**DOI:** 10.3389/fphys.2020.615690

**Published:** 2021-02-18

**Authors:** Edem Allado, Mathias Poussel, Simon Valentin, Antoine Kimmoun, Bruno Levy, Duc Trung Nguyen, Cécile Rumeau, Bruno Chenuel

**Affiliations:** ^1^EA 3450 DevAH-Développement, Adaptation et Handicap, Régulations cardio-respiratoires et de la motricité, Université de Lorraine, Nancy, France; ^2^Explorations Fonctionnelles Respiratoires et de l’Aptitude à l’Exercice, Centre Universitaire de Médecine du Sport et Activité Physique Adaptée, CHRU-Nancy, Nancy, France; ^3^Département de Pneumologie, CHRU-Nancy, Nancy, France; ^4^Médecine Intensive et Réanimation Brabois, CHRU-Nancy, Nancy, France; ^5^INSERM U1116, Université de Lorraine, Nancy, France; ^6^ORL et Chirurgie Cervico-Faciale, CHRU-Nancy, Nancy, France; ^7^INSERM U1254-IADI, Université de Lorraine, Nancy, France

**Keywords:** coronavirus disease-19, respiratory physiology, control of breathing, hypoxemia, respiratory failure

## Abstract

The growing coronavirus disease (COVID-19) crisis has stressed worldwide healthcare systems probably as never before, requiring a tremendous increase of the capacity of intensive care units to handle the sharp rise of patients in critical situation. Since the dominant respiratory feature of COVID-19 is worsening arterial hypoxemia, eventually leading to acute respiratory distress syndrome (ARDS) promptly needing mechanical ventilation, a systematic recourse to intubation of every hypoxemic patient may be difficult to sustain in such peculiar context and may not be deemed appropriate for all patients. Then, it is essential that caregivers have a solid knowledge of physiological principles to properly interpret arterial oxygenation, to intubate at the satisfactory moment, to adequately manage mechanical ventilation, and, finally, to initiate ventilator weaning, as safely and as expeditiously as possible, in order to make it available for the next patient. Through the expected mechanisms of COVID-19-induced hypoxemia, as well as the notion of silent hypoxemia often evoked in COVID-19 lung injury and its potential parallelism with high altitude pulmonary edema, from the description of hemoglobin oxygen affinity in patients with severe COVID-19 to the interest of the prone positioning in order to treat severe ARDS patients, this review aims to help caregivers from any specialty to handle respiratory support following recent knowledge in the pathophysiology of respiratory SARS-CoV-2 infection.

## Introduction

The growing coronavirus disease (COVID-19) crisis has stressed worldwide healthcare systems probably as never before, requiring a tremendous increase of the capacity of intensive care units to handle the sudden increase of patients in critical status. In many countries, innovative solutions have been found to change the routine hospital organization and cope with limited resources, leading to massive task-shifting with suspension of elective medical and surgical procedures and reassignment of volunteers (Aziz et al., 2020; Meschi et al., 2020; Xie et al., 2020b). If lung infection resulting from severe acute respiratory syndrome coronavirus 2 (SARS-CoV-2) has been shown to encompass various clinical features, the most serious presentation is worsening arterial hypoxemia, eventually leading to acute respiratory distress syndrome (ARDS) promptly needing mechanical ventilation (Guan et al., 2020 Wu and McGoogan, 2020). The systematic recourse to intubation of every patient suffering from hypoxemia may be difficult to sustain and may not be deemed appropriate for all patients. Then, it is essential that caregivers have solid knowledge of physiological principles to properly interpret arterial oxygenation, to intubate at the satisfactory moment, to adequately manage mechanical ventilation, and, finally, to begin weaning from the ventilator, as safely and as expeditiously as possible, in order to make it available for the next patient.

## COVID-19-Related Hypoxemia, Interpretation of Blood Oxygen Levels, and the Concept of “Silent Hypoxemia”

### COVID-19-Related Hypoxemia and Suspected Physiopathological Mechanisms

Hypoxemia is a defining feature of COVID-19. Viral respiratory infection has been shown to cause interstitial pneumonia, leading to a reduction in lung capacity and evolving in some patients to ARDS and respiratory failure. The typical imaging characteristics of COVID-19 pneumonia are non-specific, including peripheral ground-glass opacities with or without consolidation (Bernheim et al., 2020; Lang et al., 2020). They reflect diffuse alveolar injury associated to interstitial thickening, greatly altering gas exchange. In that context, four basic mechanisms of hypoxemia can be discussed: hypoventilation, diffusion impairment, shunt (i.e., hypoventilated areas of the lung are hyperemic), and ventilation-perfusion inequality. However, the most important cause by far is ventilation-perfusion mismatch, resulting from blood perfusing lung regions that have either limited or no ventilation [i.e., regions with low ventilation-perfusion ratios *.V*_A_/*Q.* ratios) or intraparenchymal shunt, respectively], as Gattinoni et al. have reported in their cohort of COVID-19 patients with ARDS (Gattinoni et al., 2020c). They observed a shunt fraction around ~0.5 [i.e., venous to arterial shunt estimated by the shunted blood flow/total blood flow ratio (*.Q*_s_/*.Q*_T_ ratio) of 50%] and a large alveolar-to-arterial oxygen gradient (P_A_O_2_-P_a_O_2_ gradient), enhanced by impaired hypoxic vasoconstriction (Gattinoni et al., 2020c). In addition, COVID-19 is often associated to coagulopathy, providing microemboli which could divert lung perfusion to regions with low *.V*_A_/*Q.* ratios (Altemeier et al., 1998; Connors and Levy, 2020). Two major different phenotypes of COVID-19-associated ARDS have been described and probably involve different pathophysiological mechanisms: COVID-19 pneumonia type L depicted by high compliance (i.e., low elastance), low ventilation-to-perfusion ratio, and low recruitability, and COVID-19 pneumonia type H characterized by low compliance (i.e., high elastance), high right-to-left shunt (i.e., the hypoventilated areas of the lung are hyperemic), and high recruitability, analogous to what is experienced in common acute respiratory distress (Gattinoni et al., 2020a).

Therefore, in addition to the CT scan evaluation, the response to oxygen therapy can be helpful to distinguish the two phenotypes. The delivery of raised FIO_2_ would increase PaO_2_ and oxygen saturation in the L phenotype when ventilation-to-perfusion ratio mismatch drives hypoxia, avoiding or delaying the recourse to intubation and mechanical ventilation with satisfactory levels of arterial oxygenation by oxygen therapy. At the opposite, when hypoxia is mainly determined by a shunt, in H phenotype, a modest enhancement in oxygen saturation is expected by the delivery of high FIO_2_, often requiring earlier invasive ventilator assistance (Gattinoni et al., 2020a).

The underlying physiopathology has not been fully elucidated but partly due to the SARS-CoV-2 infecting the host recognizing the angiotensin-converting enzyme 2 (ACE-2) receptor as a specific target (Hoffmann et al., 2020; Lu et al., 2020). It is a membrane-bound aminopeptidase expressed on many human cells (respiratory tract, lung, heart, arteries, veins, kidney, and intestines; Hamming et al., 2004). More particularly, the ACE-2 receptor is located in alveolar epithelial cells and vascular endothelium, and when SARS-CoV-2 binds to it, a reduction in intracellular ACE-2 protein activity is provided, resulting in a marked immune response with hyperinflammatory syndrome and widespread endothelial dysfunction (Connors and Levy, 2020; Mehta et al., 2020; Polidoro et al., 2020; Zhang et al., 2020). Physiologically, ACE-2 is a vasodepressor, at the opposite of the homologous enzyme ACE-1 acting as a vasoconstrictor, and both proteins form the oxygen-sensitive renin-angiotensin system (Hampl et al., 2015). Histopathologically, recent works have emphasized the development of alveolar and interstitial exudative inflammation characterized by macrophage and monocyte predominance and associated to focal respiratory epithelial desquamation, hemorrhage, and type 2 pneumocyte proliferation (Tian et al., 2020; Xu et al., 2020).

Hypoxemia has been shown to be an independent prognostic factor for the severe form of COVID-19 (Wei et al., 2020) and associated with in-hospital mortality (Xie et al., 2020a).

### Interpretation of Blood Oxygenation From Pulse Oximetry, Caution, and Limits

The assessment of oxygen saturation in the arterial blood by pulse oximetry should be carefully interpreted. Indeed pulse oximetry provides an estimate of the arterial oxygen saturation (SpO_2_) and is not a direct measurement, as CO-oximeters are able to do (SaO_2_). By definition, oxygen saturation is the percentage of hemoglobin-binding sites occupied by oxygen, varying according to the arterial PO_2_, as stipulated by the oxyhemoglobin dissociation curve. The difference between the two methods is not negligible, reaching as much as ±4% (Tobin, 1990).

The peculiar sigmoidal shape of the oxyhemoglobin dissociation curve involves several important features. In the higher range of partial pressures, the upper part of the curve is flat, impeding a significant decline in oxygen saturation when PO_2_ starts to drop. In contrast, the steeper portion of the dissociation curve markedly enhances the carriage of oxygen in the lungs (on-loading) and oxygen delivery to the tissues (off-loading). As lung injury progresses, leading to further impairment of gas exchange, PO_2_ may fall on the steep part of the dissociation curve (from 20 to 60mmHg), allowing noticeable changes in the measured oxygen saturation with small changes in PO_2_. In this context, the natural variability of ventilation due to physiological acts as talking, laughing, or breath holding may change the alveolar PO_2_, thereby inducing similar variations in PaO_2_. Then, oxygen saturation monitoring should be observed for at least several minutes. Moreover, the position of the dissociation curve itself can be modified by the patient’s acid-base status. Acidemia shifts it rightward and alkalemia in the opposite way. In the early course of COVID-19 pneumonia, numerous patients begin to hyperventilate in order to compensate for their collapsing PaO_2_. The hyperventilation consequently generates a respiratory alkalosis, shifting the dissociation curve to the left (increasing hemoglobin’s oxygen affinity to facilitate oxygen loading) such that the predictable decrease in oxygen saturation with a falling PaO_2_ will be dampened and, in some cases, prevented (Hamilton et al., 2004). In addition, with respect to the alveolar gas equation, the decreased alveolar CO_2_ partial pressure (PАCO_2_) will lead to a comparable increase in alveolar oxygen partial pressure (PAO_2_). These combined mechanisms are able to improve SaO_2_ in hypocapnic hypoxic stimulation compared with an isocapnic or hypercapnic hypoxia. In contrast, a right shift in oxygen dissociation (decreasing hemoglobin’s oxygen affinity to facilitate oxygen unloading) is expected with fever, an obvious clinical feature in COVID-19, leading to noticeable desaturation without any change in the chemosensitive drive of breathing.

Some important practical limits of pulse oximetry also need to be known. Movements of the digits (shivering patient, for example), avoiding to identify an adequate pulse signal, or bright artificial light as observed in an operating room can induce false low readings (Schnapp and Cohen, 1990; Sinex, 1999).

The pulse oximeter uses two different wavelengths to estimate oxygen saturation, generated by two light-emitting diodes, but both wavelengths of light are similarly absorbed by hemoglobin in arterial blood, capillary, venous blood, and other soft tissues. Then, it is necessary to distinguish the pulsatile signal of arterial blood flow in order to limit the signal-to-noise ratio and dispense a valid result (Sinex, 1999). Therefore, factors that are able to limit pulsatile blood flow in the digits, such as hypotension and use of vasoconstrictor agent as well as the presence of peripheral vascular disease or Raynaud’s phenomenon, may worsen the signal-to-noise ratio, resulting in an inaccurate estimation of arterial oxygen saturation. Chilblains have been increasingly recognized in association with COVID-19 (Bouaziz et al., 2020; Gottlieb and Long, 2020; Tosti et al., 2020), and peripheral vascular disease has been found to be associated with the usual comorbidities in patients suffering from severe COVID-19, such as diabetes and coronary artery disease (Du et al., 2020; Wu and McGoogan, 2020). It is also important to know that pulse oximeters dispense misleading results in front of either carboxyhemoglobinemia or methemoglobinemia since they are not able to distinguish these dyshemoglobinemias from oxygenated and deoxygenated hemoglobin. If carboxyhemoglobinemia is involved in heavy smokers or individuals using grills or heaters in enclosed spaces, it has been demonstrated that methemoglobinemia can result from the use of some drugs, including chloroquine (Rizvi et al., 2012).

Other important sources of artifact need to be cited, such as nail polish and increased skin pigmentation, especially if the real oxygen saturation is diminished (Bickler et al., 2005; Sutcu Cicek et al., 2011).

Furthermore, it has been demonstrated that large SpO_2_ to SaO_2_ differences exist in patients in critical condition with mediocre reproducibility of SpO_2_, specifically in shocked patients with low cardiac output or under high doses of vasopressor. In hemodynamically unstable patients, the detection limit of the sensor is most often exceeded (Van de Louw et al., 2001).

**Figure 1:**
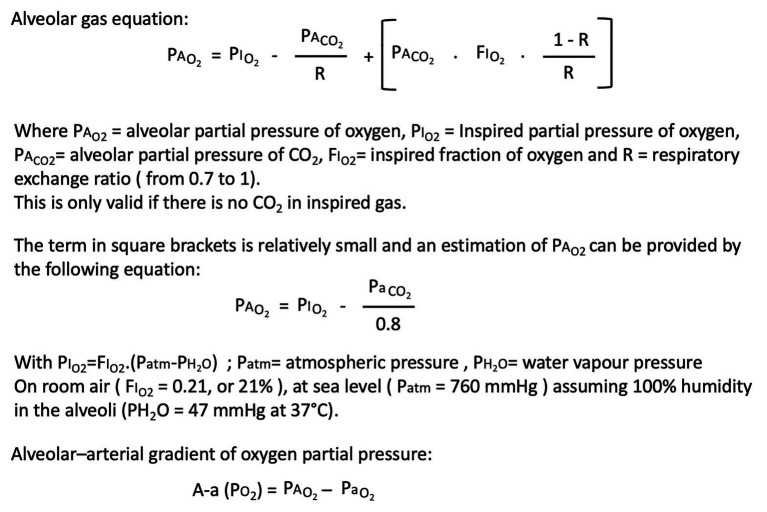
Useful toolkit to interpret oxygenation in an appropriate way.

In addition to interpretation of blood oxygenation by pulse oximetry, to correctly assess the real efficacy of pulmonary gas exchange, it is required to know the fraction of inspired oxygen (FIO_2_) in order to adequately calculate the P_A_O_2_-P_a_O_2_ gradient using the alveolar gas equation (cf. Figure 1). Then, if interpretation of blood oxygenation with supplemental oxygen is straightforward when a patient is breathing room air or is intubated, it is clearly problematic when a nasal cannula is used to deliver oxygen since the inspiratory fraction of oxygen is difficult to estimate. For example, depending on the effective patient’s minute ventilation (more specifically tidal volume patient’s demand), when a nasal cannula or a face mask is used to deliver pure oxygen flow rate at 2l/min, FIO_2_ can vary from 24 to 35% (Bazuaye et al., 1992). Therefore, the severity of hypoxemia cannot be assessed by the level of supplemental oxygen delivery. In practice, peculiar attention on the level of gas exchange impairment is recommended when high F_i_O_2_ is used to treat hypoxemia according to a simple target level on pulse oximetry, given the flatness of the upper portion of the dissociation curve (Bickler et al., 2017).

**Figure 2:**
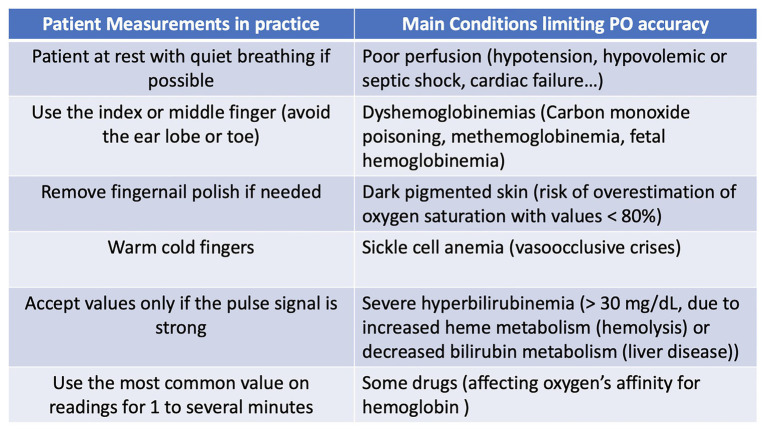
Blood oxygenation assessment with pulse oximetry (PO).

A synthesis is proposed in Figure 2 in order to present a practical assessment of blood oxygenation using pulse oximetry and limitations.

### Concept of “Silent Hypoxemia”

In one of the first largest studies on the clinical characteristics of coronavirus in China, shortness of breath has been reported in only 18.7% of 1,099 hospitalized patients with COVID-19 pneumonia, despite hypoxemia commonly requiring supplemental oxygen (41%) and abnormal results on CT scans (86.2%; Guan et al., 2020). Numerous reports worldwide have described a subset of patients with severe hypoxemia presenting no obvious respiratory difficulties or dyspnea, leading to abundant coverage in media with sensational headlines such as “happy hypoxia” or, more conventionally, “silent hypoxemia” (Couzin-Frankel, 2020; Levitan, 2020; Tobin et al., 2020b). However, in contrast to media’s assertion, this questioning discrepancy is not really defying biology since fundamentals in respiratory physiology can account for most of it, with the specific effect of SARS-CoV-2 on control of breathing or chemoreceptors excepted.

Then, knowledge of the putative mechanisms involved in the genesis of dyspnea, basics of control of breathing, ventilatory response to hypoxia, and the role of PCO_2_ is necessary to address the mystery.

### Dyspnea and Control of Breathing

Dyspnea is a highly multidimensional subjective experience needing careful assessment. It shows tremendous variability in regards to cultural and linguistic features and affective and cognitive factors (Anonymus, 1999; Parshall et al., 2012). The neurophysiologic mechanisms that give rise to the perception of dyspnea are incompletely understood, but the sensation of dyspnea probably results from a mismatch between efferent motor commands from the central nervous system (CNS) to the respiratory system and afferent sensory inputs (e.g., expected airflow, cage movements) from the respiratory system to the CNS (Adler and Janssens, 2019). It increases as inputs from receptors increase, and the central nervous system perceives that respiratory muscles cannot match the inputs and maintain adequate ventilation (Laviolette et al., 2014).

Chemoreceptors are certainly involved in the sensation of dyspnea, rising respiratory output and subsequently activating respiratory afferences, associated to corollary discharges and direct projections from chemoreceptors to forebrain structures (notably the limbic system, also underlying the genesis of pain sensation; Banzett et al., 2000; Evans et al., 2002; Buchanan and Richerson, 2009). The insular cortex appears to play a crucial role since it has been demonstrated that insular lesions are associated with a blunted perception of dyspnea (Schon et al., 2008).

With the lung injury due to SARS-CoV-2, numerous sources of stimulation of sensory receptors may gather information and feed it to the central controller, from inflammation of the respiratory tract and lungs to hypoxemia, leading to dyspnea (Tobin, 2020). However, the experience of the subjective sensation of breathlessness is not systematic, depending on the patient and circumstances and with great similarity to pain sensation (Lansing et al., 2009).

### Ventilatory Response to Hypoxia and Dyspnea

In healthy humans, the ventilatory response to partial pressure of arterial oxygen (PaO_2_) is hyperbolic (Rebuck and Campbell, 1974). Reducing the partial pressure of arterial oxygen (PaO_2_) from its normal value to 60mmHg has a marginal effect on pulmonary ventilation (*.V*_E_) and PaCO_2_ (Forster and Dempsey, 1981). Nevertheless, further reducing PaO_2_, from about 60 to 30mmHg, provides a progressive increase in *.V*_E_ following an exponential pattern (hyperbolic curve) and a decrease in PaCO_2_ (Forster and Dempsey, 1981). In contrast, the relationship between ventilation and arterial oxygen saturation (SaO_2_) is linear (Rebuck and Campbell, 1974). Physiologically, in human subjects, the increase in ventilation occurs primarily because of a rise of tidal volume and only a small increase in the frequency of breathing (Reynolds and Milhorn, 1973; Bender et al., 1987). If tachypnea is one of the most important clinical indicators of respiratory distress, it could be without proportion to severe hypoxemia. Moreover, in COVID-19 patients, tachypnea would be more elicited by stimulation of lung receptors (pulmonary stretch, irritant, and J receptors) due to lung inflammation than by the hypoxic stimulus and therefore would not be the cornerstone of the intubation decision (Tobin, 2020).

It has been demonstrated that the level of hypoxia corresponding to the perception of air hunger in healthy subjects matches with the sharp increase of minute ventilation but far from all the subjects have complained as a strong increase in air hunger with a fall end-tidal oxygen partial pressure below 60mmHg (Moosavi et al., 2003). Dyspnea often occurs when PaO_2_ declines below 40mmHg (Manning and Schwartzstein, 1995). Like the large variability of the resting respiratory drive, there is a great between-subject and within-subject variability of ventilatory response to hypoxia in healthy subjects (Sahn et al., 1977; Tobin et al., 1988; Matsuzawa et al., 1989). It has been demonstrated that the ventilatory response to hypoxia is decreased by half in elderly healthy people (Kronenberg and Drage, 1973; Peterson et al., 1981). The decrease is even more pronounced in patients suffering from diabetes (Nishimura et al., 1989; Weisbrod et al., 2005), who not only presented an impaired perception of sensory input from organs but also demonstrated an increased threshold for the perception of respiratory sensations has been (O’Donnell et al., 1988). Since diabetes is among the most frequently reported comorbidities and the median age is easily over 60years in patients infected with COVID-19, it is not so surprising to observe numerous cases of “silent hypoxemia” (Grasselli et al., 2020; Huang et al., 2020; Richardson et al., 2020).

Furthermore, hypoxia is also well known to depress ventilation at the central nervous system level, possibly masking unpleasant sensations (Berry et al., 1989).

### Modulation of the Hypoxic Ventilatory Response by CO_2_

In the absence of isocapnia, the ventilatory response to hypoxia is severely attenuated by hypocapnia associated with hyperventilation. This attenuation is due to an effect on the peripheral chemoreceptors (carotid body essentially) as well as to reduced drive from the central chemoreceptors (Lahiri and DeLaney, 1975; Fitzgerald and Dehghani, 1982; Moore et al., 1984). It has been demonstrated that moderate hypocapnia, corresponding to PaCO_2_ values from 5 to 10mmHg below eucapnia, flattened the hypoxic response, suggesting that a minimum level of CO_2_ is required to generate the hypoxic ventilatory response (Jounieaux et al., 2002; Corne et al., 2003; Wilson and Teppema, 2016). In order to elicit a valuable rise in ventilation, severe hypoxia must be associated to baseline PaCO_2_ that exceeds 39mmHg (Moosavi et al., 2003). Since hypoventilation is uncommon with COVID-19, hypoxemia accompanied by a normal alveolar-to-arterial oxygen gradient and increase in PaCO_2_ is highly unlikely, especially in the early phase of lung injury. In the great majority of severe cases, hypoxemia is accompanied by an increased alveolar-to-arterial oxygen gradient reflecting either ventilation-perfusion mismatch or intrapulmonary shunting and the compensatory ventilatory response to hypoxemia, leading to noticeable hypocapnia (Tobin, 2020).

Consequently, knowledge of the accompanying PaCO_2_ is imperative to assess the severity of the respiratory failure associated to hypoxemia, another reason to claim that isolated monitoring of SaO_2_ is insufficient to guide clinical decisions.

Taken together, it would not be so astonishing that many COVID-19 patients face hypoxemia and rapid respiratory failure without evidence of dyspnea.

## Lessons From High Altitude and Aviation Physiology: Are the Similarities Between COVID-19 Ards and High-Altitude Pulmonary Edema Relevant?

The common clinical pattern of COVID-19 lung injury is based upon a noticeable imbalance between relatively well-preserved lung compliance and a severely impaired pulmonary gas exchange, resulting in hypoxemia without corresponding signs of dyspnea or respiratory distress. Since the physiological characteristics of the hypocapnic ventilatory response to hypoxia have been extensively investigated in high altitude physiology and aviation medicine, learnings from them could be helpful in order to better manage the COVID-19 pandemic.

Beyond the apparent similarity between the COVID-19 silent hypoxemia and the non-lethal high altitude-induced hypoxemia associated to respiratory alkalosis, even allowing climbers to exercise in ascent despite very low levels of PaO_2_, some authors have advocated parallelism between COVID-19 acute respiratory distress syndrome and high-altitude pulmonary edema (HAPE), with great amplification *via* social media (Solaimanzadeh, 2020).

With the first descriptions of the clinical features of severe COVID-19 pneumonia, a debate has emerged on the development of typical ARDS or not, allowing specific and important clinical implications (Gattinoni et al., 2020b,c). Most of the patients with severe COVID-19 pneumonia meet the criteria that define internationally the ARDS [ARDS Berlin definition: acute onset of hypoxemia assessed by the PaO_2_/FIO_2_ ratio ≤300mmHg in a ventilated patient with a positive end-expiratory pressure (PEEP) of at least 5cmH_2_O and bilateral lung infiltrates not fully explained by heart failure or volume overload (Force et al., 2012)], but unusual presentations exist (Gattinoni et al., 2020b,c). The main difference is relatively well-preserved lung mechanics with maintenance of a relatively high respiratory system compliance (close to the normal value of 50ml/cm H_2_O), in contrast to typical severe ARDS (Gattinoni et al., 2020b,c). For some authors, the hypothesis for such hypoxemia associated to compliant lungs could be a hypoxic vasoconstriction (Gattinoni et al., 2020c). HAPE and ARDS are a non-cardiogenic form of pulmonary edema characterized by diffuse bilateral opacities on chest imaging caused by an imbalance in Starling forces, thus inducing fluid accumulation in the interstitial and alveolar spaces. However, the pathogenesis of such pulmonary edema is radically different between the two entities. HAPE is related to an excessive hypoxia-mediated increase in pulmonary vascular resistance or hypoxic pulmonary vasoconstriction increasing microvascular pressure and leading to a substantial increase in pulmonary artery pressure with overperfusion of some regions of the lung, elevated pulmonary capillary hydrostatic pressure, and leakage of fluid into the alveolar space (Swenson and Bartsch, 2012). Consequently, HAPE is a life-threatening condition that is favorably influenced (often reversed) by oxygen therapy, exposure to hyperbaric environment (using portable hyperbaric chambers), or descent/evacuation to lower altitude and, finally, very unusually needs intensive care (Swenson and Bartsch, 2012; Strapazzon et al., 2020). Since hypoxic vasoconstriction is the fundamental pathogenesis mechanism in HAPE, increasing the alveolar PO_2_ decreases pulmonary artery pressure, allowing the resolution of alveolar and interstitial edema and full recovery within hours to a few days of exposure. Distinctly, the underlying pathophysiological mechanisms in ARDS due to COVID-19 involve multi-organ viral-mediated inflammatory responses leading in the lung to genesis of alveolar epithelial inflammation and dysfunction of surfactant and alveolar fluid clearance, finally leading to alveolar collapse and/or filling and marked ventilation-perfusion mismatch (Gattinoni et al., 2020a). Therefore, in marked contrast to HAPE, the delivery of supplemental oxygen in COVID-19 pneumonia may increase oxygen availability but will not be able to counteract the underlying inflammation or lung injury (Luks and Swenson, 2020; Strapazzon et al., 2020). This major distinction has crucial clinical implications since drugs well known to inhibit hypoxic pulmonary vasoconstriction—acetazolamide, systemic vasodilators like calcium channel blockers, or phosphodiesterase-5 inhibitors—are not only inappropriate but also expected to worsen ventilation/perfusion mismatch by raising prefusion blood flow to poorly and/or nonventilated lung regions, exacerbating hypoxemia and provoking hypotension in COVID-19 patients (Archer et al., 2020; Brugger et al., 2020; Luks and Swenson, 2020; Strapazzon et al., 2020).

## On the Interest of Prone Positioning in COVID-19 Pneumonia, Not Only to Improve Gas Exchange but Also as a Strategy to Delay or Avoid Mechanical Ventilation

Prone positioning, i.e., when a patient is repositioned from supine position to lie on their front, has been used for more than 45years to improve oxygenation in patients with acute respiratory failure and more specifically with ARDS (Guerin, 2014). Historically, in the 1970s, Mellins observed that children suffering from advanced cystic fibrosis spontaneously position themselves on their hands and knees to improve their ventilation, while Bryan hypothesized that, in acute respiratory failure with consequent impairment of functional residual capacity and enhancement of dependent airway closure, the prone position might recruit and stabilize the dependent lung (Bryan, 1974; Mellins, 1974). Since then, numerous randomized controlled trials and meta-analyses have demonstrated a conclusive and important mortality reduction using prone positioning early and for a prolonged time in subjects with severe ARDS (Abroug et al., 2008; Alsaghir and Martin, 2008; Guerin et al., 2013; Beitler et al., 2014; Hu et al., 2014; Lee et al., 2014; Bloomfield et al., 2015; Munshi et al., 2017). Nowadays, prone positioning is used not only as an efficient treatment in case of life-threatening hypoxemia but also in the prevention of ventilatory-induced lung injury (VILI; Chiumello and Brioni, 2016; Guerin, 2017; Mitchell and Seckel, 2018).

The main underlying physiologic mechanism for the ensuing improvement in patients’ oxygenation with prone position is the decrease in intrapulmonary shunting, but an improvement of ventilatory mechanics is also involved (Gattinoni et al., 2013; Guerin et al., 2014). Prone positioning provides reduction in intrapulmonary shunt (*.Q*_s_/*.Q*_T_), variation in lung ventilation (*.V*_A_), and lung perfusion (*.Q*) distribution with improved *.V*_A_/*Q.* matching. By recruiting dorsal regions which have a larger number of alveolar units and by obtaining an increase in chest wall elastance, better ventilation to the perfused lung is provided, improving the ventilation/perfusion ratio and allowing a more homogeneous distribution of ventilation. This leads to a decrease in lung strain and, consecutively, reduction of VILI, reducing the risk of right heart failure (Gattinoni et al., 2013; Guerin et al., 2014; Ruste et al., 2018). The improvement of oxygenation in ARDS patients during a prone session is observed in ~75% of the cases and sometimes intense (Guerin, 2014). The positive oxygenation response is commonly defined as an improvement in PaO_2_ by 20% or an increase in the PaO_2_/FIO_2_ ratio by 20mmHg (Guerin, 2014). It has been demonstrated that prone positioning reduced relative shunt fraction by about 30% and improved PaO_2_/FIO_2_ ratio by 34–62%, with a variable temporal response (from an immediate response to a continued response for up to 24h; Kallet, 2015; Scholten et al., 2017).

Additional data are also important to note concerning the drainage of secretions which improves when prone, with material in the dorsal lung traveling more easily to open airways. Nevertheless, no significant reduction in the incidence of ventilator-associated pneumonia has been observed in a recent prospective study cohort of patients with severe ARDS (Ayzac et al., 2016). Major improvements in thoraco-abdominal compliance were particularly observed in patients with higher body mass index (Kallet, 2015).

During the COVID-19 pandemic, the use of prone positioning was proposed not only in ARDS patients requiring mechanical ventilation, as it is internationally recommended (Alhazzani et al., 2020; Wilson et al., 2020), but also in order to avoid or delay the recourse to intubation in the dramatic context of limited resources and capacity of intensive care units (Chad and Sampson, 2020; Elharrar et al., 2020; Sartini et al., 2020; Villarreal-Fernandez et al., 2020).

Innovative solutions have been found worldwide to cope with limited resources and to include the prone positioning in the management of patients requiring mechanical ventilation, even at the surge of the outbreak, resulting in the emergence of prone teams (Doussot et al., 2020; Kimmoun et al., 2020; Settembre et al., 2020).

In COVID-19 patients, the Surviving Sepsis campaign recommends a trial of prone positioning in mechanically ventilated patients who meet the moderate-to-severe ARDS definition (Alhazzani et al., 2020). Periods of 12–16h are suggested, based upon evidence for non-COVID ARDS (Alhazzani et al., 2020).

In conscious non-ventilated COVID-19 patients, it is expected that the underlying mechanism leading to an improvement in oxygenation is analogous, but only few studies evaluated the benefits of the prone position and no clear recommendations have emerged (Elharrar et al., 2020; Sartini et al., 2020). Short-term improvements of oxygenation are observed in such patients, but further studies are needed to clarify the real benefit, particularly on mortality.

## Physiological Basis for Ventilatory Support

If the initial message from the Chinese medical teams at the surge of the outbreak was to intubate early, the current ventilatory approach is to delay intubation if it clinically appears safe and feasible (Alhazzani et al., 2020). Currently, any therapy that could prevent intubation and mechanical ventilation (MV) or enhance MV weaning without further deterioration is welcome. Regrettably, “safe” lung-protective ventilation does not really exist; thus, ventilatory support needs to be individualized as the best compromise among respiratory mechanics, recruitability, gas exchange, and hemodynamics to minimize VILI and to ensure adequate oxygenation when arterial hypoxemia is refractory to oxygen therapy.

The spectrum of therapies and the different lung support which have been proposed to the management of ARDS with critical hypoxemia (i.e., severe ARDS, with PaO_2_/FIO_2_ <100mmHg) encompass the delivery of oxygen therapy by high-flow nasal cannula (HFNC) system and non-invasive positive pressure ventilation (NIPPV). In severe COVID-19 patients, these therapies should only be used in selected patients with hypoxemic respiratory failure and who are closely observed for early detection of further deterioration (Pfeifer et al., 2020).

With oxygen flow rates that can reach 60–80L per minute, HFNC systems can more accordingly ensure the ventilatory demands of patients with respiratory distress and respiratory failure compared to the standard nasal cannula (Suffredini and Allison, 2020). They are able to reduce dead space, raise the end-expiratory lung volume, improve compliance, and reduce the work of breathing, resulting in improvement of pulmonary gas exchange (Suffredini and Allison, 2020). There is limited data to promote or refute the use of HFNC in SARS-CoV-2 and in ARDS patients; the failure rate has been found to be relatively high (Messika et al., 2015). However, it has been proposed to be combined with prone positioning (Colla et al., 2020; Suffredini and Allison, 2020; Villarreal-Fernandez et al., 2020). Decisions to continue HFNC treatment might depend on the results of periodic clinical assessments and repeated biological measurements corroborating clinical stability or improvement (Suffredini and Allison, 2020).

The use of NIPPV with a pressure support tailored to ensure a tidal volume between 7 and 10ml/kg and a PEEP set between 2 and 10cm H_2_O could also lessen the intrapulmonary shunt and diminish the work of breathing, but just as the HFNC, NIPPV is associated with a high risk of failure and associated risks of a delayed start of invasive mechanical ventilation (Evans, 2001). The clinical result of the use of NIPPV needs to be carefully assessed, and if, following the first few hours, no significant improvement in pulmonary gas exchange is observed, it should be ceased and invasive mechanical ventilation should be initiated (Evans, 2001). More specifically, the magnitude of oxygenation disturbance is a predictor of NIPPV failure, and a PaO_2_/FIO_2_ ratio <150mmHg is described as the decisive threshold for increased mortality (Bellani et al., 2017). However, some very recent works have emphasized the interest on non-invasive strategies in COVID-19, especially in order to avoid intubation (Brusasco et al., 2020; Oranger et al., 2020; Tobin et al., 2020a).

### Invasive Mechanical Ventilation

The decision to intubate mainly relies on the clinical judgment of the critical care physician but is also based upon combined features such as level of hypoxemia, respiratory distress, increased work of breathing, fatigue, and gas exchange (Tobin, 2020). In the peculiar context of the COVID-19 pandemic, the most appropriate timing for the intubation of hypoxic patients with severe lung injury is not well known and also depends on the local capacity for mechanical ventilation.

The main objective of mechanical ventilation is to lessen work and the oxygen cost of breathing, allowing oxygen stores to be redirected to vulnerable tissue beds (Tobin et al., 2012). In patients in acute respiratory distress, it has been demonstrated that the oxygen cost of breathing is enhanced to as much as 50% of total oxygen consumption (Field et al., 1984).

The basic principles of the assist-control ventilation are based upon the delivery of a breath under positive pressure provided by the ventilator, either triggered by the inspiratory effort achieved by the patient (pressure or flow triggered) or, independently, if such an effort is not performed within a preselected time period.

The main challenge for the physician then is to cycle the rhythm of the ventilator in synchrony with the patient’s central respiratory rhythm while improving gas exchange. Three critical points have been identified: triggering (cycling on), post-trigger inflation, and inspiration-expiration switchover (cycling off; Tobin et al., 2012; Tobin, 2018).

The two most common modes used for mechanical ventilation are pressure-controlled ventilation (PCV), using a predetermined inflation pressure applied for a predetermined inflation time, and volume-controlled ventilation (VCV), using a predetermined volume.

With PCV, the delivered volume varies according to the properties of the respiratory system and also to the patient’s effort and the inspiratory flow displays a decelerating shape; in VCV, the delivered volume is maintained constant, independently of the patient’s effort, while the airway pressure is non-uniform and the inspiratory flow has a fixed shape.

It is important to note that the amount of active work performed by a patient in volume-cycled assist-control crucially relies on the sensitivity of the trigger and inspiratory flow settings. Despite optimal selected settings, it has been established that patients actively perform about a third of the work carried out by the ventilator during passive conditions (Marini et al., 1985). Pressure support can efficiently decrease the work of inspiration, but the level of inspiratory muscle unloading appears highly labile, with a coefficient of variation reaching up to 96% among patients (Jubran et al., 1995).

If mechanical ventilation is a valid life-saving intervention, it can also enhance lung injury and, through VILI, contribute to multi-organ failure in patients with ARDS (Slutsky and Ranieri, 2013). The major determinant of VILI is the genesis of non-physiologic stress (tension) and strain (deformation), which relies not only on the size of the delivered tidal volume but also on the amount of lung resting volume (Gattinoni et al., 2012).

Therefore, the most common strategy to minimize VILI is low tidal volume (*V*_T_) ventilation. A *V*_T_ from 4 to 8ml/kg of predicted body weight is recommended in mechanically ventilated adults with COVID-19 and ARDS (Alhazzani et al., 2020). Along with low *V*_T_ ventilation, lower airway pressure use [i.e., plateau pressure (*P*_plat_) ≤30cmH_2_O] is a lung-protective strategy (Petrucci and De Feo, 2013).

### Ventilator Weaning

Considering the side effects of mechanical ventilation and, additionally, the limitation of the intensive care resources during the COVID-19 pandemic, it is critical to get patients off the ventilator at the earliest possible time.

Since a delayed initiation of the weaning process has recurrently been observed, weaning predictor tests have been developed (Yang and Tobin, 1991; Tobin and Jubran, 2006). Among the physiological measurements that can alert a physician at initiating the weaning process, the level of rapid shallow breathing, quantified by frequency of breathing-to-*V*_T_ ratio (fb/*V*_T_), has been shown to be the best predictor of weaning outcome (Yang and Tobin, 1991; Tobin and Jubran, 2006). Synchronized mandatory ventilation is not recommended (Brochard et al., 1994).

Several approaches are used to manage weaning: from the use of a T-tube circuit allowing bouts of spontaneous breathing trials to the gradual reduction in the level of ventilator assistance (Tobin et al., 2012). Almost invariably, weaning failure arises within the first hour of attempted spontaneous breathing (Tobin, 2018).

## Conclusion

In COVID-19 lung injury, as observed in many other respiratory diseases, control of breathing is the cornerstone of the clinical presentation, from dyspnea to respiratory failure, not only explaining symptoms but also allowing appropriate levels of physiological compensations in order to maintain efficient spontaneous ventilation. However, when overwhelmed, a patient critically requires ventilator assistance, which also greatly involves the key elements of the control of breathing.

A clear view of COVID-19-related hypoxemia needs an appropriate interpretation of blood oxygenation from pulse oximetry, keeping in mind cautions and limits of accuracy. The role of the position of the dissociation curve associated to changes of the patient’s acid-base status or hyperventilation-related hypocapnia, as well as the calculation of the P_A_O_2_-P_a_O_2_ gradient using the alveolar gas equation, is crucial to assess the real efficacy of pulmonary gas exchange. The participation of ventilatory response to hypoxia in the genesis of dyspnea and its modulation by CO_2_ can help to explain that many COVID-19 patients face hypoxemia and rapid respiratory failure without evidence of dyspnea.

When mechanical ventilation is decided in critical COVID-19 patients, the usual strategies to tailor it are involved, based upon the basis of respiratory physiology to lessen work and the oxygen cost of breathing. The safe discontinuation of mechanical ventilation needs a careful assessment of physiological parameters (level of rapid shallow breathing) in order to warn a physician that a ventilated patient might be able to come off the ventilator in order to make it available for the next patient in such a peculiar context of the COVID-19 pandemic.

## Author Contributions

EA, MP, AK, CR, and BC contributed to conception of this work: literature search, drafting, writing, and critical review of the text. SV, DN, and BL contributed to literature search, writing, and critical review of the final document. All authors contributed to the article and approved the submitted version.

### Conflict of Interest

The authors declare that the research was conducted in the absence of any commercial or financial relationships that could be construed as a potential conflict of interest.
